# SARS-CoV-2 wildlife surveillance in Ontario and Québec

**DOI:** 10.14745/ccdr.v48i06a02

**Published:** 2022-06-09

**Authors:** Janet E Greenhorn, Jonathon D Kotwa, Jeff Bowman, Laura Bruce, Tore Buchanan, Peter A Buck, Christina M Davy, Antonia Dibernardo, Logan Flockhart, Marianne Gagnier, Aaron Hou, Claire M Jardine, Stephane Lair, L Robbin Lindsay, Ariane Massé, Pia K Muchaal, Larissa A Nituch, Angelo Sotto, Brian Stevens, Lily Yip, Samira Mubareka

**Affiliations:** 1 Wildlife Research and Monitoring Section, Ontario Ministry of Northern Development, Mines, Natural Resources and Forestry, Peterborough, ON; 2 Sunnybrook Research Institute, Toronto, ON; 3 Centre for Food-borne, Environmental and Zoonotic Infectious Diseases, Public Health Agency of Canada; 4 Department of Biology, Carleton University, Ottawa, ON; 5 National Microbiology Laboratory, Public Health Agency of Canada, Winnipeg, MB; 6 Ministère des Forêts, de la Faune et des Parcs du Québec, Québec, QC; 7 Canadian Wildlife Health Cooperative, Ontario-Nunavut, Department of Pathobiology, University of Guelph, Guelph, ON; 8 Canadian Wildlife Health Cooperative, Québec, Saint-Hyacinthe, QC; 9 Department of Laboratory Medicine and Pathobiology, University of Toronto, Toronto, ON

**Keywords:** SARS-CoV-2, wildlife, surveillance, Ontario, Québec

## Abstract

**Background:**

Severe acute respiratory syndrome coronavirus 2 (SARS-CoV-2), the virus responsible for the coronavirus disease 2019 pandemic, is capable of infecting a variety of wildlife species. Wildlife living in close contact with humans are at an increased risk of SARS-CoV-2 exposure and, if infected, have the potential to become a reservoir for the pathogen, making control and management more difficult. The objective of this study is to conduct SARS-CoV-2 surveillance in urban wildlife from Ontario and Québec, increasing our knowledge of the epidemiology of the virus and our chances of detecting spillover from humans into wildlife.

**Methods:**

Using a One Health approach, we leveraged activities of existing research, surveillance and rehabilitation programs among multiple agencies to collect samples from 776 animals from 17 different wildlife species between June 2020 and May 2021. Samples from all animals were tested for the presence of SARS-CoV-2 viral ribonucleic acid, and a subset of samples from 219 animals across three species (raccoons, *Procyon lotor*; striped skunks, *Mephitis mephitis*; and mink, *Neovison vison*) were also tested for the presence of neutralizing antibodies.

**Results:**

No evidence of SARS-CoV-2 viral ribonucleic acid or neutralizing antibodies was detected in any of the tested samples.

**Conclusion:**

Although we were unable to identify positive SARS-CoV-2 cases in wildlife, continued research and surveillance activities are critical to better understand the rapidly changing landscape of susceptible animal species. Collaboration between academic, public and animal health sectors should include experts from relevant fields to build coordinated surveillance and response capacity.

## Introduction

The severe acute respiratory syndrome coronavirus 2 (SARS-CoV-2) is responsible for the global coronavirus disease 2019 (COVID-19) pandemic and has been maintained through human-to-human transmission. However, humans are not the only species susceptible to infection. Over the course of the current pandemic, a range of domestic and wild animal species have been reported to either be naturally infected with SARS-CoV-2 or susceptible to the virus in experimental infections ((1–4)). As of April 30, 2022, 36 countries have reported positive SARS-CoV-2 cases in 23 different animal species to the World Organisation for Animal Health ((5)). Other species have been identified as potential hosts based on sequence analysis of the host cell receptor of SARS-CoV-2, angiotensin 1 converting enzyme 2 and predicted binding affinity ((6,7)).

Many wild animal species, such as raccoons, skunks and bats, thrive in the ecological overlap with humans and are thus at an increased risk of being exposed to SARS-CoV-2 ((8)). Several peri-domestic species have been experimentally shown to become infected with and shed SARS-CoV-2 ((9,10)). SARS-CoV-2 infection has also been reported in wild or free-ranging animals that have been naturally exposed, including American mink (*Neovison vison*) in Spain ((11)) and, more recently, white-tailed deer (*Odocoileus virginianus*) in multiple locations across North America ((12–16)). In Ontario, this includes identification of a probable case of deer-to-human viral transmission ((16)). Infection in animals can result in mild to severe symptoms of respiratory disease up to and including death via interstitial pneumonia (e.g. mink) ((17,18)). Other species do not show clinical signs of infection (e.g. skunks) ((9,10)) or show only mild and transient symptoms in some individuals, such as elevated temperature (e.g. white-tailed deer) ((19)).

The concept of One Health recognizes that human and animal health are interdependent ((20)). The spillover of virus from humans or domestic animals into wildlife is concerning not only due to the possible deleterious effects on wildlife, but because these wild populations have the potential to act as reservoirs for SARS-CoV-2. Pathogens that have an animal reservoir are inherently more difficult to control and the spread of SARS-CoV-2 through animal populations could further contribute to the development of variants of concern (VoCs), potentially undermining the efficacy of countermeasures such as antivirals and vaccines ((21,22)). As such, there have been calls for increased surveillance at the human-wildlife interface ((23)). Urban areas around the world have been a particular area of concern and focus ((24–26)). The higher density of both human and some peri-urban wildlife species populations in urban centres can lead to more frequent human-animal contact and increased potential for disease transmission. Additionally, people who have close contact with wildlife, such as biologists, rehabilitators, and hunters and trappers, may be at higher risk of being exposed to the virus and of facilitating its spread among wildlife. The impact of SARS-CoV-2 infection on wildlife health is not fully understood. Early detection of any spillover is therefore critical to preventing and addressing these concerns.

Given the risk of reverse-zoonotic SARS-CoV-2 transmission and our lack of knowledge of the virus in local wildlife, there was an urgent need to elucidate the epidemiology of the virus at the human-wildlife interface to help wildlife management and public health officials better communicate risk and plan management strategies. We therefore conducted SARS-CoV-2 surveillance in wildlife across Ontario and Québec, with a major focus on the southern regions of both provinces. These areas have high human population densities and include major urban centres such as Toronto and Montréal. Between spring 2020 and spring 2021, incidences of COVID-19 peaked in Montréal and the surrounding regions in early January 2021, with rates exceeding 400 cases per 100,000 population in Montréal and Laval ((27)). Incidences between spring 2020 and spring 2021 in the Greater Toronto Area peaked in April 2021, with case rates in the City of Toronto and Peel also exceeding 400 per 100,000 population ((27)).

## Methods

Many experts have recommended a One Health approach for animal SARS-CoV-2 testing, which balances concerns for both human and animal health and is based on knowledge of experts in both fields ((28,29)). As such, our work was conducted through consultation and cooperation among a wide variety of agencies: the Public Health Agency of Canada; the Ontario Ministry of Northern Development, Mines, Natural Resources and Forestry (NDMNRF); *le Ministère des Forêts, de la Faune et des Parcs du Québec*; the Canadian Wildlife Health Cooperative (CWHC); the Ontario Ministry of Agriculture, Food, and Rural Affairs; the Canadian Food Inspection Agency; the Western College of Veterinary Medicine; the Granby Zoo; the National Microbiology Laboratory (NML) of the Public Health Agency of Canada; and Sunnybrook Research Institute. All samples for testing were collected between June 2020 and May 2021 through pre-existing partnerships or over the course of other research, surveillance or rehabilitation work (**Table 1**).

**Table 1:** Metadata for 776 animals from Ontario and Québec screened for severe acute respiratory syndrome coronavirus 2

Species	Sampling agency	Sample source	Sample location(s)	Dates of collection	Number of individuals sampled	Types of samples tested	Test performed^a^
Raccoon (*Procyon lotor*)	CWHC	Rabies surveillance (Québec samples), post-mortem exam	Southern Ontario, Southern Québec	Aug 2020–Feb 2021	11	Respiratory tissue	PCR
			Southern Québec	Nov–Dec 2020	68	Respiratory tissue, rectal swab	
			Southern Ontario, Southern Québec	Oct 2020–June 2021	15	Respiratory and intestinal tissue	
			Southwestern Québec	Jan 2021	3	Nasal swab	
			Southern Québec	Jan–June 2021	54	Nasal and rectal swabs	
	NDMNRF and CWHC	Rabies surveillance, post-mortem exam	Hamilton, Ontario	Dec 2020	1	Oral and rectal swabs, respiratory and intestinal tissue	
	NDMNRF	Rabies surveillance	Southwestern Ontario	June 2020–Jan 2021	100	Oral and rectal swabs	
		Rabies seroprevalence study	Oakville, Ontario	Sept–Oct 2020	141	Oral and rectal swabs	
						Sera	Antibody
Total raccoons sampled					393	-	
Striped skunk (*Mephitis mephitis*)	CWHC	Rabies surveillance (Québec samples), post-mortem exam	Southern Québec	Jan–June 2021	66	Nasal swab	PCR
			Southern Ontario, Southern Québec	July–Dec 2020	55	Respiratory tissue	
			Southern Ontario, Southwestern Québec, Saint-Félicien, Québec	Oct 2020–Apr 2021	9	Respiratory and intestinal tissue	
	NDMNRF	Rabies surveillance, rabies seroprevalence study	Southwestern Ontario	Sept 2020–May 2021	104	Oral and rectal swabs	
		Rabies seroprevalence study	Oakville, Ontario	Sept–Oct 2020	36	Oral and rectal swabs	
						Sera	Antibody
Total skunks sampled					270	-	
American mink (*Neovision vison*)	CWHC	Post-mortem exam	Thornhill, Ontario	July 2020	1	Respiratory tissue	PCR
	NDMNRF	Registered fur harvesters, roadkill, rabies surveillance	Southern Ontario	Fall 2020–Spring 2021	42^b^	Oral and rectal swabs, lung and intestinal tissue	
						Cardiac blood or Nobuto strips	Antibody
Total mink sampled					43	-	
Big brown bat (*Eptesicus fuscus*)	Granby Zoo	Rehabilitation program	Southwestern Québec	Nov 2020–Mar 2021	15	Oral swabs	PCR
					2	Guano	
					15	Oral swabs and guano	
Total big brown bats sampled					32	-	
Hoary bat (*Lasiurus cinerus*)	CWHC	Post-mortem exam	Etobicoke, Ontario	Dec 2020	1	Respiratory and intestinal tissue	PCR
American marten (*Martes americana*)	CWHC	Post-mortem exam	Sainte-Anne-de-Bellevue, Québec	Nov 2020	1	Respiratory and intestinal tissue	PCR
Fisher (*Pekania pennanti*)	CWHC	Post-mortem exam	Western Québec	May 2021	2	Respiratory and intestinal tissue	PCR
American black bear (*Ursus americanus*)	CWHC	Post-mortem exam	Northern Ontario	Sept 2020	2	Respiratory tissue	PCR
			Killaloe, Ontario	Oct 2020	1	Respiratory and intestinal tissue	
Total black bears sampled					3	-	
Atlantic white-sided dolphin (*Lagenorhynchus actus*)	CWHC	Post-mortem exam	Carleton-sur-Mer, Québec	June 2021	1	Intestinal tissue	PCR
			Sept-Îles, Québec	March 2021	1	Respiratory and intestinal tissue	
Total Atlantic white-sided dolphins sampled					2	-	
Harbour porpoise (*Phocoena phocoena*)	CWHC	Post-mortem exam	La Montée, Québec	Dec 2020	1	Respiratory and intestinal tissue	PCR
Harbour seal (*Phoca vitulina*)	CWHC	Post-mortem exam	Matane, Québec	Dec 2020	1	Respiratory and intestinal tissue	PCR
Coyote (*Canis latrans*)	CWHC	Post-mortem exam	Saint-Alexandre-d'Iberville, Québec	April 2021	1	Respiratory and intestinal tissue	PCR
Eastern wolf (*Canus lupus lycaon*)	CWHC	Post-mortem exam	Algonquin Provincial Park, Ontario	Oct 2020	1	Respiratory tissue	PCR
			Southern and central Ontario		4	Respiratory and intestinal tissue	
Total eastern wolves sampled					5	-	
Grey fox (*Urocyon cinereoargenteus*)	CWHC	Post-mortem exam	Châteauguay, Québec	Dec 2020	1	Respiratory and intestinal tissue	PCR
Red fox (*Vulpes vulpes*)	CWHC	Post-mortem exam	Mercier, Québec	Jan 2021	1	Nasal and rectal swabs	PCR
			Southwestern Québec	Nov–Dec 2020	4	Respiratory tissue, rectal swabs	
			Southern, Ontario	July–Oct 2020	5	Respiratory tissue	
			Dunham, Québec	Dec 2020	1	Respiratory and intestinal tissue	
Total red foxes sampled					11	-	
Virginia opossum (*Didelphis virginiana*)	CWHC	Post-mortem exam	Bolton-Est, Québec	June 2021	1	Nasal and rectal swabs	PCR
			Southern Ontario	July–Oct 2020	2	Respiratory tissue	
			Southwestern Ontario, Saint-Jean-sur-Richelieu, Québec	Oct 2020, March 2021	3	Respiratory and intestinal tissue	
Total Virginia opossums sampled					6	-	
White-tailed deer (*Odocoileus virginianus*)	CWHC	Post-mortem exam	London, Ontario, Southwestern Québec	Oct–Dec 2020	3	Respiratory and intestinal tissue	PCR

Abbreviations: CWHC, Canadian Wildlife Health Cooperative; NDMNRF, Northern Development, Mines, Natural Resources and Forestry; PCR, polymerase chain reaction; -, not applicable

^a^ All PCR testing was performed at Sunnybrook Research Institute and all antibody testing was performed at the Public Health Agency of Canada’s National Microbiology Laboratory

^b^ Due to the condition of the carcasses, we were unable to collect lung tissue or cardiac blood from one individual, cardiac blood from a further two individuals and rectal swabs from two individuals. In cases where we could not collect cardiac blood, we instead submitted a Nobuto strip soaked in fluid from the chest cavity for antibody testing

### Raccoons and skunks

**Figure 1:**
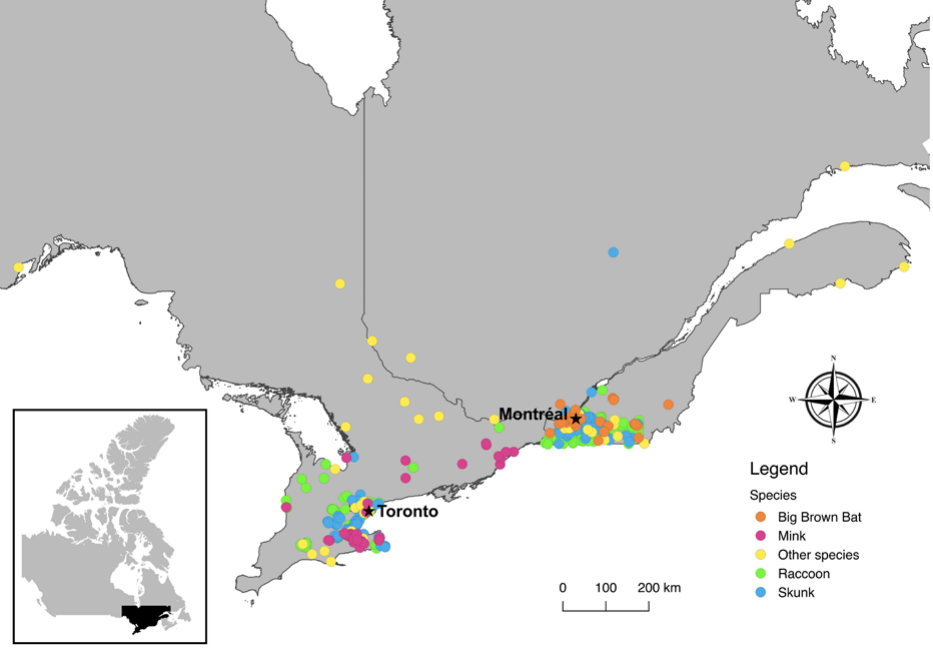
Original locations of animals submitted for severe acute respiratory syndrome coronavirus 2 testing June 2020–May 2021 (N=776)

Raccoons (*Procyon lotor*) and striped skunks (*Mephitis mephitis*) are peri-domestic species that are good candidates for reverse-zoonotic disease surveillance due to their high density in urban areas and their frequent close contact with people, pets and refuse. They are also subject to ongoing rabies surveillance operations in both Ontario and Québec, making them easy to sample. In Ontario, wildlife rabies surveillance and testing are conducted by the NDMNRF on roadkill, animals found dead for other reasons, and wildlife that were sick or acting strangely. Submissions are received mainly from southwestern Ontario, and most animals received by the program and subsequently sampled and tested for SARS-CoV-2 came from urban centres within this region or had a case history of close contact with people (**Figure 1**). In Québec, a similar wildlife rabies surveillance program is coordinated by *le Ministère des Forêts, de la Faune et des Parcs du Québec* and testing and other post-mortem examinations are performed by the Québec CWHC. As was the case in Ontario, animals sampled by the Québec CWHC for SARS-CoV-2 testing came mainly from urban areas (Figure 1). The Ontario CWHC laboratory also contributed a small number of raccoon and skunk samples from animals submitted to them for post-mortem examination. Carcasses were sampled using a combination of oral, nasal, and rectal swabs, respiratory tissue and intestinal tissue (Table 1). Swabs were stored in individual 2 mL tubes with ~1 mL of universal transport medium (UTM; Sunnybrook Research Institute) and 30–60 mg tissue samples were stored dry in tubes.

Additionally, samples were collected from live raccoons and skunks during an annual seroprevalence study conducted by the NDMNRF in Oakville, Ontario to assess the effectiveness of rabies vaccine baiting (NDMNRF Wildlife Animal Care Committee Protocol #358). Animals were captured in live traps and transported to a central processing station where they were anaesthetized. Oral and rectal swabs were collected for polymerase chain reaction (PCR) testing. Blood was drawn from the brachiocephalic vein and 0.2–1.0 mL of sera was collected for antibody testing. Following reversal and successful recovery, animals were returned to their point of capture and released.

### Mink

Instances of SARS-CoV-2 infection in mink have already been identified in multiple countries, including Canada, and infected farmed mink have proven capable of passing the virus to naive conspecifics, humans and companion animals ((17,30–33)). At the time of writing no mink farm outbreaks have been reported in Ontario or Québec, but mink farms in Ontario have previously been shown to act as points of infection for other viruses (e.g. Aleutian mink disease), which can spread to wild mink populations ((34)).

The majority of mink carcasses we sampled for SARS-CoV-2 testing were submitted to the NDMNRF by licensed fur harvesters through a collaboration with the Ontario Fur Managers Federation. The NDMNRF staff collected oral and rectal swabs, lung tissue and intestinal tissue from the carcasses, as well as cardiac blood samples via cardiac puncture for antibody testing. If blood could not be obtained from the heart, fluid was collected from the chest cavity on a Nobuto filter strip (Advantec MFS, Inc, Dublin, California, United States [US]). Nobuto strips were allowed to air dry, then placed in individual coin envelopes.

### Big brown bats

Bats are known carriers of coronaviruses ((35–37)). As such, concerns have been raised over the possible susceptibility of North American bats to SARS-CoV-2 ((38)). Species such as the big brown bat (*Eptesicus fuscus*) frequently roost in buildings, which brings them into close contact with people and increases the likelihood of SARS-CoV-2 exposure. Big brown bat oral swabs and guano samples for SARS-CoV-2 PCR testing were collected by staff at the Granby Zoo, which runs a rehabilitation program over the winter to care for bats that have been disturbed during their hibernation. Guano samples were stored dry in 2 mL tubes.

### Other species

Other samples for SARS-CoV-2 PCR testing were obtained opportunistically through the Ontario and Québec regional CWHC laboratories, which receive a wide variety of wildlife species for post-mortem examination (Table 1). Animals were selected for sampling based on potential for SARS-CoV-2 infection. This could be due to urban habitat, human contact or to predicted species susceptibility based on prior research. The number and type of samples collected varied by carcass and depended on carcass condition (Table 1).

### Ribonucleic acid extraction

Ribonucleic acid (RNA) extraction and PCR testing were performed at the Sunnybrook Research Institute in Toronto, Ontario. All swab, tissue and guano samples were stored at -80°C prior to testing. For oral, rectal or nasal swab samples, RNA extractions were performed using 140 µL of sample via the QIAmp viral RNA mini kit (Qiagen, Mississauga, Ontario) or the Nuclisens EasyMag using Generic Protocol 2.0.1 (bioMérieux Canada Inc., St-Laurent, Québec) according to manufacturer’s instructions. The RNA from guano samples (80 mg) were extracted via the QIAmp viral RNA mini kit and eluted in 40 µL in containment level 3 at the University of Toronto. Tissue samples were thawed, weighed, minced with a scalpel, and homogenized in 600 µL of lysis buffer using the Next Advance Bullet Blender (Next Advance, Troy, New York, US) and a 5 mm stainless steel bead at 5 m/s for 3 minutes. The RNA from 30 mg tissue samples was extracted via the RNeasy Plus Mini kit (Qiagen, Mississauga, Ontario) or the Nuclisens EasyMag using Specific Protocol B 2.0.1; RNA was eluted in 50 µL. All extractions were performed with a positive and negative control. Extraction efficiency between kits was assessed through comparison of positive extraction controls.

### Severe acute respiratory syndrome coronavirus 2 polymerase chain reaction analysis

Real-time polymerase chain reaction (RT-PCR) was performed using the Luna Universal Probe One-Step RT-qPCR kit (NEB). Two gene targets were used for SARS-CoV-2 RNA detection: the 5’ untranslated region (UTR) and the envelope (E) gene ((39)). This assay was adapted from the Shared Hospital Labs from The Research Institute of St. Joseph Hamilton for use in animals. The cycling conditions were as follows: one cycle of denaturation at 60°C for 10 minutes then 95°C for 2 minutes followed by 44 amplification cycles of 95°C for 10 seconds and 60°C for 15 seconds. Quantstudio 3 software (Thermo Fisher Scientific Inc., Waltham, Massachusetts, US) was used to determine cycle thresholds (Ct). All samples were run in duplicate and samples with Cts less than 40 for both gene targets in at least one replicate were considered positive.

### Antibody testing

Antibody testing was performed on cardiac blood, chest cavity fluid and serum samples at the NML in Winnipeg, Manitoba. All samples were stored at -20°C prior to testing. Cardiac blood samples were collected onto Nobuto filter strips by saturating the length of the strip with 100 µl of blood. To obtain the 1:9 dilution required for testing, saturated Nobuto strips were cut into 4–5 pieces and placed into a 2 mL tube containing 360 µl phosphate buffered saline pH 7.4 containing 0.05% Tween 20 and eluted overnight at 4°C. Nobuto strips collected from chest cavity fluid were processed in the same way, whereas serum samples were diluted 1:9 with Sample Dilution Buffer. Samples were mixed by vortexing and tested using the GenScript cPass™ SARS-CoV-2 Neutralization Antibody Detection Kit (GenScript US, Inc. Piscataway, New Jersey, US) according to the manufacturer’s protocol.

Briefly, 60 µl of a sample was added to 60 µl HRP-conjugated RBD solution and incubated at 37°C for 30 minutes. A 100 µl aliquot of the mixture was transferred to the ELISA microwell test plate and incubated at 37°C for 15 minutes. Microwells were washed four times with 260 µl wash buffer then 100 µl TMB substrate was added to each well. Following a 20-minutes incubation in the dark at room temperature, 50 µl of Stop Solution was added to each well. Absorbance was read immediately at 450 nm.

Each assay plate included positive and negative controls that met required quality control parameters. Percentage inhibition was calculated for each sample using the following equation:

% inhibition = (1- optical density sample / optical density negative control) x 100%

Samples with greater than or equal to 30% inhibition were considered positive for SARS-CoV-2 neutralizing antibodies.

## Results

We tested 776 individual animals from 17 different wildlife species for SARS-CoV-2. These animals were collected primarily from urban areas in southern Ontario and Québec between June 2020 and May 2021 (Table 1). We found no evidence of SARS-CoV-2 viral RNA in any of the tested samples and no evidence of neutralizing antibodies in a subset of 219 individuals (141 raccoons, 36 striped skunks, 42 mink).

## Discussion

Our study did not detect any spillover of SARS-CoV-2 to wildlife in Ontario and Québec. Raccoons and skunks were the most commonly tested species. Results from experimental studies have suggested these species may be susceptible to SARS-CoV-2, but the lack of and low quantity of infectious virus shed by raccoons and skunks, respectively, suggest they are an unlikely reservoir for SARS-CoV-2 in the absence of viral adaptations ((9,10)). Similarly, a challenge study with big brown bats found that they are resistant to SARS-CoV-2 infection and do not shed infectious virus ((40)). Conversely, minks are susceptible to SARS-CoV-2 infection, but no evidence of SARS-CoV-2 was detected in any of the mink sampled. While this could be attributed to low effective sample size, to date SARS-CoV-2 has been infrequently detected in wild mink populations globally. It should be noted, however, that these experimental studies on raccoons, skunks and big brown bats ((9,10,40)) were conducted using parental SARS-CoV-2. The susceptibility of these species to VoCs is presently not known and may differ from susceptibility to the parental strain ((41)). Additionally, challenge studies assessing susceptibility tend to be conducted on small numbers of young, healthy individuals, so results may not be reflective of the full range of possible responses to infection in the wild.

As the pandemic progresses, new evidence is emerging on susceptible wildlife that may act as competent reservoirs for the virus. For example, white-tailed deer are now considered a highly relevant species for SARS-CoV-2 surveillance in light of their experimentally determined susceptibility as well as evidence of widespread exposure to the virus via antibody and PCR testing across North America ((12–16,19)). Continued surveillance efforts should be adaptive and include targeted testing of highly relevant species as they are identified. In Ontario and Québec, these would include mink, white-tailed deer and deer mice (*Peromyscus maniculatus*) ((9,42)). Continuing to include less susceptible species remains important given ongoing viral genomic plasticity and changing host range of VoCs.

### Limitations

There are several limitations for this study that need to be acknowledged. First, the majority of our SARS-CoV-2 testing was done by RT-PCR, which is only capable of detecting active infection. Antibody testing, which identifies resolved infection or exposure, is more likely to find evidence of SARS-CoV-2 in surveillance studies since results are less dependent on timing of sample collection. Antibody testing typically requires samples from live animals or fresh carcasses, which limited our ability to use it; however, the testing performed allowed for test validation in raccoons, skunks and mink, which may facilitate more antibody testing in future. Second, we relied on different kits for RNA extraction due to logistical challenges. Based on our extraction controls, the QIAamp RNA mini kit performed slightly better compared to the Nuclisens EasyMag (~2 Cts) for swab samples. Conversely, the Nuclisens EasyMag performed slightly better (~2 Cts) compared to the RNeasy mini plus kit for tissue samples. Third, the type of samples we collected may also have limited our ability to detect SARS-CoV-2 infection. Viral replication can vary among tissue types and therefore some tissues are more optimal for viral RNA detection than others ((1)). In the present work, animals were sampled opportunistically as a part of pre-existing programs, and we were not able to consistently collect the same sample sets. Additionally, the sample types were from live animals and carcasses and not optimized; certain sample types were sometimes unavailable (e.g. tissue samples from live animals) or were not sufficient for collection.

### Conclusion

A One Health approach is critical to understanding and managing the risks of an emerging zoonotic pathogen such as SARS-CoV-2. We leveraged activities of existing research, surveillance, and rehabilitation programs and expertise from multiple fields to efficiently collect and test 1,690 individual wildlife samples. The absence of SARS-CoV-2-positive wildlife samples does not exclude spillover from humans to Canadian wildlife, given the limitations cited above. Continued research in this area is both important and pressing, particularly as novel VoCs emerge. Public and animal health sectors should continue to work collaboratively with academic and government partners to help prevent the spread of SARS-CoV-2 from people to wildlife, monitor for spillover, and address any issues should they arise. There is an urgent need for a coordinated wildlife surveillance program for SARS-CoV-2 in Canada. This approach will help protect the health of both Canadians and wildlife, now and in the future.
